# Tagging Single Nucleotide Polymorphisms in the *IRF1* and *IRF8* Genes and Tuberculosis Susceptibility

**DOI:** 10.1371/journal.pone.0042104

**Published:** 2012-08-06

**Authors:** Shiping Ding, Tao Jiang, Jianqin He, Beibei Qin, Shuangyan Lin, Lanjuan Li

**Affiliations:** 1 State Key Laboratory for Diagnosis and Treatment of Infectious Diseases, the First Affiliated Hospital of Medical College, Zhejiang University, Hangzhou, China; 2 The National Education Base for Basic Medical Sciences, School of Medicine, Zhejiang University, Hangzhou, China; Kunming Institute of Zoology, Chinese Academy of Sciences, China

## Abstract

Genes encoding IRF1 and IRF8 protein have been proposed as candidate tuberculosis susceptibility genes. In order to elucidate whether the *IRF1* and *IRF8* variants were associated with tuberculosis susceptibility, we conducted a case-control study consisting of 495 controls and 452 ethnically matched cases with tuberculosis in a Chinese population. Seven haplotype tagging single-nucleotide polymorphisms (tagSNPs) (rs2057656; rs2706381; rs2070724; rs2070721; rs2549008; rs2549007; rs2706386) from HapMap database were analyzed, which provided an almost complete coverage of the genetic variations in the *IRF1* gene. Fifteen tagSNPs (rs12924316; rs182511; rs305080; rs2292980; rs925994; rs424971; rs16939967; rs11117415; rs4843860; rs9926411; rs8064189; rs12929551; rs10514611; rs1044873; rs6638) were observed in the *IRF8* gene. All these tagSNPs were genotyped by SNPstream genotyping and SNaPshot typing. None of the seven tagSNPs was individually associated with tuberculosis in the *IRF1* gene. In the *IRF8* gene, interestingly, we found that three tagSNPs (rs925994 and rs11117415 located in the intron region; rs10514611 located in the 3′UTR) were associated with risk of tuberculosis after Bonferroni correction. Per allele OR was 1.75 (95% CI 1.35∼2.27, *P* = 0.002), 4.75 (95% CI 2.16∼10.43, *P* = 0.002) and 3.39 (95% CI 1.60∼7.20, *P* = 0.015) respectively. Luciferase reporter gene assay showed that the construct that contained the non-risk allele C of rs10514611 showed significantly higher luciferase activity than did the risk T allele (*P*<0.01), which implied rs10514611 was a potential functional SNP site. Our results indicated that the *IRF8* gene might participate in genetic susceptibility to tuberculosis in a Chinese population.

## Introduction

The infectious disease tuberculosis (TB) is still widespread. According to the World Health Organization, two billion people are infected with the causative bacillus *Mycobacterium tuberculosis* [1]. Epidemiological data has shown that only one tenth of *Mycobacterium tuberculosis* infections will finally become clinically apparent. Other studies have demonstrated that the prevalence and incidence of TB are significantly higher in identical twins compared to fraternal twins [2]. Additionally, its prevalence is significantly different among different races and ethnic groups [3]. These suggest a possible influence of genetic susceptibility in the development of TB [4]–[7].

The immune response to TB is primarily cell mediated, and involves a variety of T cells, macrophages, and cytokines. Studies have shown that T helper (Th) cell subsets (Th1 verse Th2) and their cytokines are essential to prevent the invasion of *Mycobacterium tuberculosis* [8]–[10]. The interferon regulatory factor (IRF) family is a group of newly identified transcription factors and plays an important role in the regulation of Th cell differentiation [11]–[13]. Among the IRF family, IRF1 and IRF8 proteins are implicated in resistance to intracellular infection [14]–[16]. Genes encoding IRF1 and IRF8 protein have been proposed as candidate TB susceptibility genes. The *IRF1* gene (Gene ID: 3659) is located at chromosome 5q31.1. It comprises ten exons [17]–[18]. The gene product is a protein of 325 amino acids. The *IRF8* gene (Gene ID: 3394) is located at chromosome 16q24.1. It comprises nine exons. The gene product is a protein of 426 amino acids [19].

Several recent findings suggest that the *IRF1* gene is an important risk factor for some allergic diseases [20]–[22]. Vollstedt *et al.*, in contrast, found no association of the *IRF1* gene with pulmonary TB [23]. However, whether more common *IRF8* variants are also associated with TB has not been investigated. Different population may have different genetic associations with disease. To elucidate the role of the *IRF1/IRF8* in TB susceptibility, we tested the association of seven tagSNPs of the *IRF1* and fifteen tagSNPs of the *IRF8* with risk of TB in a Chinese case-control study. We attempted to identify sufficient SNPs to tag all the common haplotypes across the *IRF1* and *IRF8* genes.

## Materials and Methods

### Study Population

The case-control study included 452 histopathologically confirmed TB patients and 495 TB-free controls. TB group: Han Chinese with TB were selected from the Hangzhou Municipal Red-Cross Hospital based on the diagnostic criteria of TB. Sputum culture, tuberculin skin test, clinical X-ray examination, and pathological examinations were performed for all cases. Patients with positive sputum culture results were confirmed as sputum smear-positive TB, which required two consecutive positive smears. Patients with negative sputum culture results but positive by the purified protein derivative test (PPD test), X-ray examination, and clinical manifestations consistent with the diagnostic criteria of TB were confirmed as sputum culture-negative TB. All patients responded to anti-mycobacterial treatment and were followed up. Exclusion criteria were: patients with pneumonia, lung cancers, or other diseases with similar clinical features; patients with hepatitis; individuals with HIV/AIDS or those who had received immuno-suppressors; and patients with other severe diseases. Control group: Healthy Han Chinese were randomly recruited from the same district as a control population. These individuals were not related to members of the TB group, with none having a history of TB as confirmed by X-ray and physical examinations and tuberculin skin test.

**Table 1:** Descriptive characteristics in the Chinese case-control study.

Index	Controlsn (%)	TB casesn (%)	*P* value
Age (years)			
Mean (± S.D.)	46±15	45±16	
<50	330 (66.7)	310 (68.6)	0.81
50–60	55 (11.1)	46 (10.2)	
>60	110 (22.2)	96 (21.2)	
Gender			
Male	253 (51)	317 (70)	<0.01
Female	242 (49)	135 (30)	
Family History			
Yes	36 (7%)	107 (24%)	<0.01
No	459 (93%)	345 (76%)	
Clinical type, no. (%)			
Pulmonary		396	
Sputum/Culture positive		240 (60.6)	
Clinical-radiological and Histology diagnosed		156 (39.4)	
Extrapulmonary TB^a^		56	
Culture-proven		18 (32.1)	
Histology diagnosed		38 (67.9)	

a Extrapulmonary TB includes lymph node, pleural, bone and renal TB.

All subjects signed informed consent forms voluntarily and the research was approved by the Medical Ethics Commission of Zhejiang University.

### SNP Identification and Selection

Using the HapMap genome browser (http://www.hapmap.org/cgi-perl/gbrowse/hapmap3r2_B36) based on the CHB+JPT population, seven tagSNPs (r^2^ coefficient cut-off of 0.85 with a minor allele frequency of 0.15), were selected to capture the *IRF1* within a 23 kb region of chromosome 5 and fifteen tagSNPs for the *IRF8* within a 23 kb region of chromosome 16.

### Genotyping

Genomic DNA was extracted from 1.5 ml of peripheral blood samples using the Puregene DNA isolation kit (Gentra Systems, Minneapolis, MN, USA). Two tagSNPs of the *IRF1* and ten tagSNPs of the *IRF8* were genotyped using the GenomeLab SNPstream Genotyping System (Beckman Coulter, Fullerton, CA). Five tagSNPs of the *IRF1* gene and five tagSNPs of the *IRF8* gene were genotyped using the SNaPshot typing (Applied Biosystems, Foster City, CA, USA). Primers were shown in Table S1 and Table S2. Repeated genotyping of >10% randomly selected samples yielded 100% identical results.

**Table 2:** Primary information for seven tagSNPs of the *IRF1* gene.

TagSNPs	Substitution	Position	Location	MAF	*P*	Rate (%)
rs2057656	C > T	131809305	3′-UTR	0.36	0.05	99.90
rs2706381	C > T	131810619	3′-UTR	0.40	0.13	98.63
rs2070724	T > C	131822072	Intron	0.33	0.66	97.67
rs2070721	C > A	131825842	Intron	0.33	0.71	97.57
rs2549008	C > T	131826853	Promoter	0.11	0.18	98.63
rs2549007	G > A	131826875	Promoter	0.34	0.64	99.90
rs2706386	T > C	131832687	5′-UTR	0.32	0.45	98.63

Position: data from Genome build 37.1; MAF, minor allele frequency; *P* value, HWE, Hardy-Weinberg equilibrium for both cases and controls; rate%: Genotyping rate.

**Table 3:** Association analysis of the *IRF1* tagSNPs with TB using logistic regression (*n* = 947).

SNP number	Genotype	Model*	Controls	Cases	OR (95% CI)	*P*
rs2057656	CC/CT/TT	Recessive	212/212/68	189/187/64	1.07 (0.73–1.55)	0.74
rs2706381	CC/CT/TT	Over-dominant	180/223/89	162/204/75	1.10 (0.84–1.43)	0.48
rs2070724	TT/TC/CC	Log-additive	218/215/48	197/189/57	1.04 (0.86–1.27)	0.67
rs2070721	CC/CA/AA	Over-dominant	210/220/50	207/191/45	0.90 (0.69–1.18)	0.45
rs2549008	CC/CT/TT	Recessive	383/106/3	352/85/4	1.97 (0.43–9.10)	0.38
rs2549007	GG/GA/AA	Over-dominant	217/223/51	197/186/58	0.81 (0.62–1.05)	0.11
rs2706386	TT/TC/CC	Over-dominant	226/219/47	212/176/53	0.76 (058–0.99)	0.054

OR, odds ratio; 95%CI, 95% confidence intervals.

*P, P*-value are for the SNPs after controlling for gender and age.

* The model with the smallest AIC value was defined as the best model for each SNP.

### Statistical Analysis

Data from the control and TB groups were compared and analyzed with SPSS 11.5 software (SPSS Inc., Chicago, IL, USA). HWE software was used to test the Hardy-Weinberg equilibrium. A χ^2^ test was performed to compare the distribution of genotypes between the TB and control groups, and the results calculated using SNPstats software (http://bioinfo.iconcologia.net/snpstats/). Based on the multivariable logistic regression method, the case-control association of genotypes in five inheritance models (codominant, dominant, recessive, overdominant, log-additive) were tested and these models were coded as follows for genotypes AA AB BB (assume B is risk allele): Dominant 0 1 1 (AA vs AB-BB); Recessive 0 0 1 (AA+AB vs BB); Additive 0 1 2 (trend test on B allele count); Overdominant 0 1 0 (AA+BB vs AB); Codominant 0 1 0 & 0 0 1 (AA vs AB & AA vs BB) and the odds ratios (OR) and 95% confidence intervals (95% CI) were given. Akaike’s information criterion (AIC) was used to choose the inheritance model that best fitted the data.

**Figure 1:**
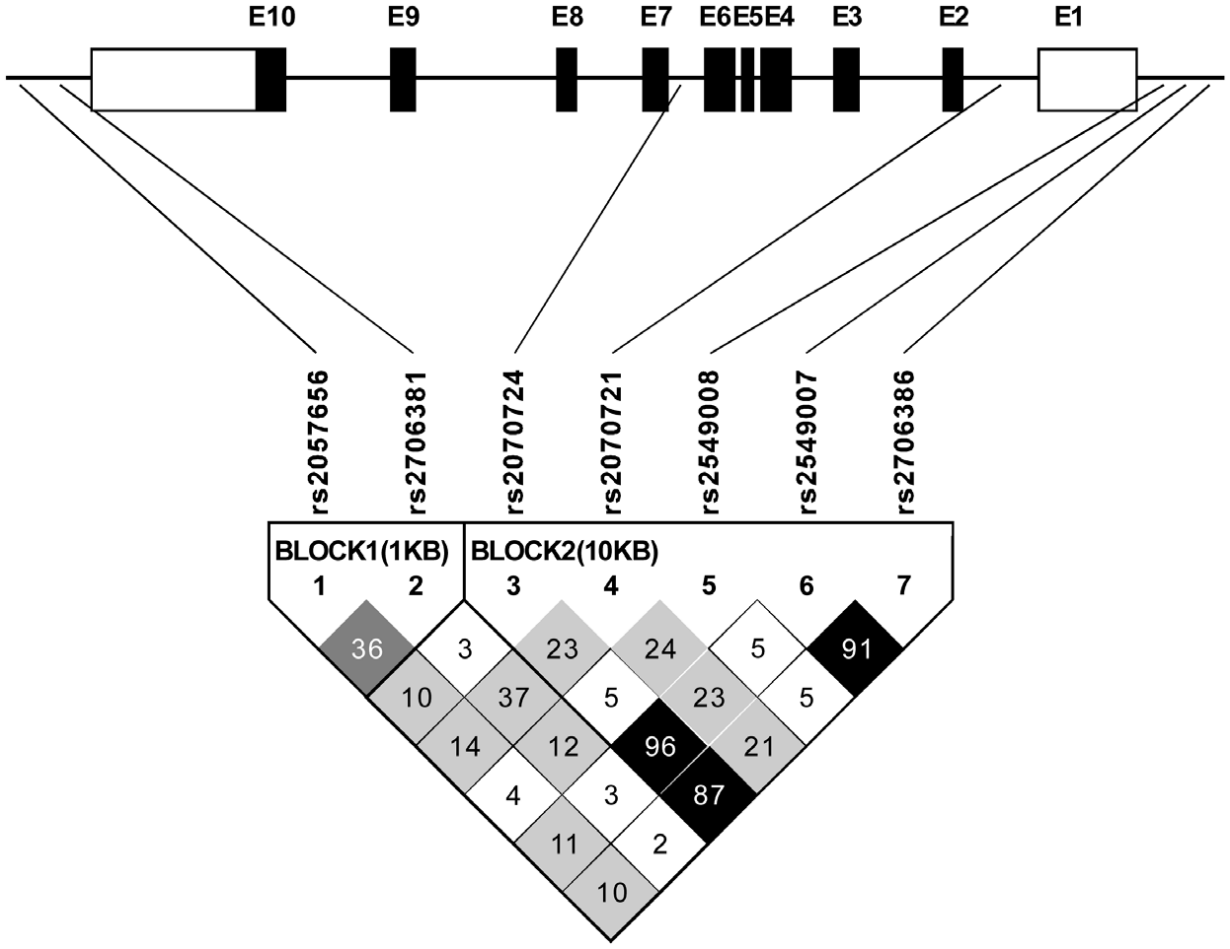
Linkage disequilibrium (LD) statistic (r^2^) for the *IRF1* gene. (Genomic organization of the *IRF1* gene, including the position of individual non-coding (open boxes) and coding (closed boxes) exons; the darker shading indicates stronger LD between SNPs).

**Table 4:** Primary information for fifteen tagSNPs of the *IRF8* gene.

TagSNPs	Substitution	Position	Location	MAF	*P* value	Rate(%)
rs12924316	C > T	85936263	Intron	0.25	0.54	98.63
rs182511	C > G	85937651	Intron	0.43	1.00	98.63
rs305080	C > T	85941774	Intron	0.31	0.19	97.36
rs2292980	T > C	85945076	Intron	0.44	0.11	97.04
rs925994	C > A	85946017	Intron	0.17	0.19	98.52
rs424971	T > C	85946450	Intron	0.49	<0.0001	98.52
rs16939967	G > T	85949473	Intron	0.78	0.63	96.94
rs11117415	A > G	85950686	Intron	0.22	0.18	97.04
rs4843860	A > G	85950921	Intron	0.29	0.94	96.73
rs9926411	A > G	85951657	Intron	0.34	0.72	98.52
rs8064189	G > T	85951796	Intron	0.45	0.64	98.31
rs12929551	C > T	85953010	Intron	0.21	0.84	98.52
rs10514611	C > T	85955242	3-UTR	0.2	0.84	97.36
rs1044873	C > T	85955671	3-UTR	0.39	0.17	96.94
rs6638	T > A	85956044	3-UTR	0.16	0.04	97.25

Position: data from Genome build 37.1; MAF, minor allele frequency; *P* value, HWE, Hardy-Weinberg equilibrium for both cases and controls; rate%: Genotyping rate.

**Figure 2:**
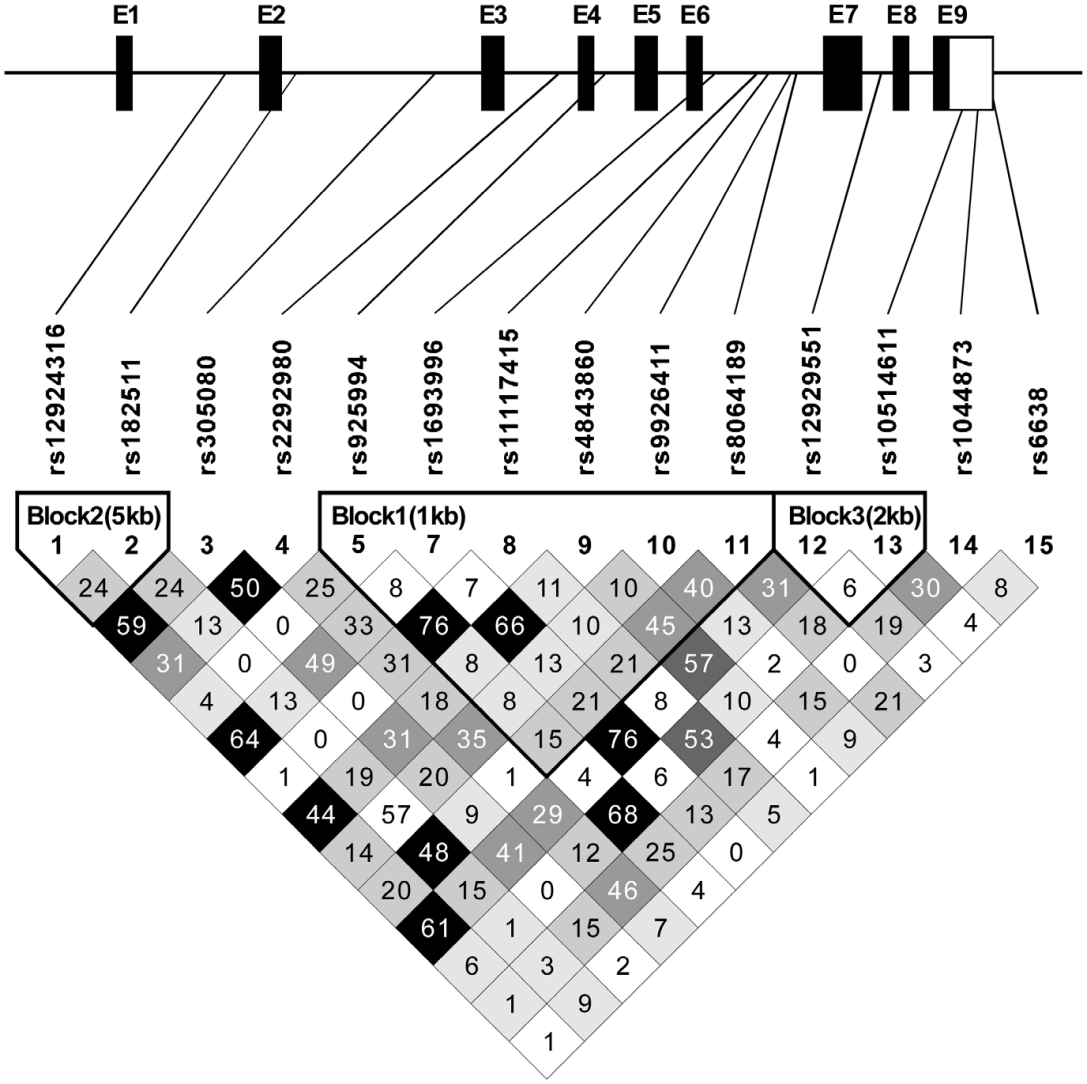
Linkage disequilibrium statistic (r^2^) for the *IRF8* gene. (Genomic organization of the *IRF8* gene, including the position of individual non-coding (open boxes) and coding (closed boxes) exons; the darker shading indicates stronger LD between SNPs).

Bonferroni corrections for multiple SNPs were performed using the formula: α = 1−(1−α′)1/n (corrected for n comparisons, α’ = 0.05, n = 22). A *P*-value less than 0.05 was regarded as statistically significant.

Using online statistical software (http://sampsize.sourceforge.net/), with the current sample size (495 controls and 452 cases) and significance threshold (0.05), we estimated that we had >65% power to detect a common risk allele (MAF>15%) with an odds ratio (OR) of 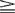
1.5.

**Table 5:** Association analysis of the *IRF8* tagSNPs with TB using logistic regression (*n* = 947).

SNP number	Genotype	Model*	Controls	Cases	OR (95% CI)	*P*	*P^adjusted^*
rs12924316	CC/CT/TT	Over-dominant	267/200/26	259/155/27	0.79(0.60–1.03)	0.081	
rs182511	CC/CG/GG	Log-additive	155/244/94	147/214/80	0.95(0.79–1.14)	0.590	
rs305080	CC/CT/TT	Recessive	225/222/33	204/191/47	1.55(0.97–2.50)	0.066	
rs2292980	TT/TC/CC	Recessive	148/250/78	123/229/91	1.41(1.00–1.99)	0.05	
rs925994	CC/CA/AA	Log-additive	345/125/6	268/157/13	1.75(1.35–2.27)	<1e-04	0.002
rs16939967	GG/GT/TT	Over-dominant	273/182/20	284/138/21	0.72(0.54–0.95)	0.018	
rs11117415	AA/AG/GG	Co-dominant	310/158/9	243/171/28	1.44(1.09–1.91)	<1e-04	0.002
rs4843860	AA/AG/GG	Dominant	219/216/41	245/161/34	0.66(0.50–0.86)	0.0034	
rs9926411	AA/AG/GG	Over-dominant	213/222/57	197/191/53	0.93(0.72–1.21)	0.61	
rs8064189	GG/GT/TT	Log-additive	132/246/112	156/207/78	0.74(0.61–0.89)	0.0027	
rs12929551	CC/CT/TT	Over-dominant	292/181/19	292/129/20	0.71(0.53–0.94)	0.015	
rs10514611	CC/CT/TT	Recessive	318/152/10	265/150/27	3.39(1.60–7.20)	0.0007	0.015
rs1044873	CC/CT/TT	Recessive	176/238/61	156/219/68	1.30(0.89–1.90)	0.18	
rs6638	TT/TA/AA	Recessive	330/137/11	318/122/3	0.28(0.08–1.04)	0.0038	

OR, odds ratio; 95%CI, 95% confidence intervals;

*P, P*-value are for the SNPs after controlling for gender and age.

*P^adjusted^*, *P*-value after Bonferroni correction accounting for multiple SNPs (correction factor = 22).

* The model with the smallest AIC value was defined as the best model for each SNP.

### Plasmid Construction and Reporter Gene Assay

PolymiRTS (http://compbio.uthsc.edu/miRSNP) was employed to search for putative SNPs that affected miRNA targeting in human. To produce pMIR-IRF8 plasmid, a fragment covering the entire 3′-UTR of the *IRF8* gene was amplified by PCR from a genomic DNA sample using the primers 5′-gga tgc ctt act ttg cac tta att -3′ and 5′-gaa cac ata ttc ctg cac caa att ct-3′ and was cloned into pMIR-REPORT vector (Ambion). The mutation on miR-330-binding sites in human *IRF8* 3′-UTR was generated by overlap PCR. HeLa cells (obtained from American Type Culture Collection) were seeded 4×10^4^/well in 24-well plates, 24 h prior to transfection. Cells were cotransfected with 0.1 µg of the following constructs: pMIR-IRF8, pMIR-REPROT together with 40 nM of miR-330-3P precursor molecule, or 40 nM of negative control no.1 using siPROT amine transfection agent (Ambion) following manufacturer’s protocol. The plasmid pRL-TK which contained the gene encoding *Renilla* luciferase (Promega, WI, USA) was cotransfected in order to normalize results. Luciferase activities were measured by dual-luciferase reporter assay system (Promega, WI, USA) 24 h after transfection. For each variant, three independent transfections were performed.

**Figure 3:**
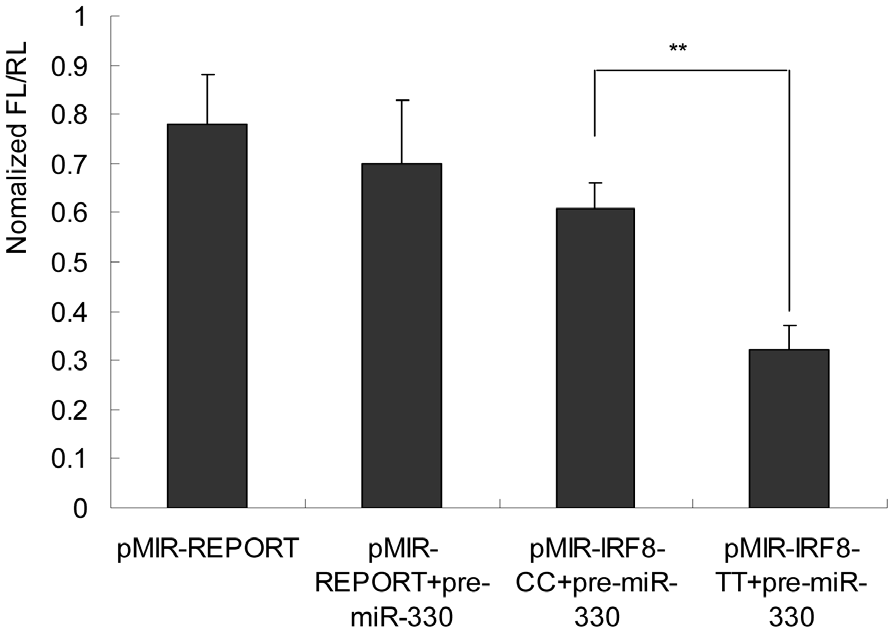
The regulation of luciferase activity by the *IRF8* 3′-UTR is dependent on miR-330. (HeLa cells were transfected with the pRL-TK containing *Renilla* luciferase gene and the indicated vectors or precursors. Bars indicated the *Firefly* luciferase activities normalized to *Renilla* luciferase activities of the cotransfected pRL-PK vector. **P<0.01).

## Results

### Epidemiological Features in the Control and TB Group

A total of 495 DNA samples in the control group (mean age: 46 years, interquartile range, from 37 to 56 years) and 452 samples in the TB group (mean age: 45 years, interquartile range, from 36 to 53 years) was extracted. The control group included 253 males (51%) and 242 females (49%), while the TB group included 317 males (70%) and 135 females (30%) (Table 1). Twenty four percent of the TB goup had a family history of TB compared to 7% of the control group. Of the cases, the TB group encompassed 396 patients with pulmonary TB and 56 patients with extrapulmonary TB (including lymph node, pleural, bone and renal TB).

### Relationship between tagSNPs of the *IRF1* Gene and TB

Table 2 showed the primary information for the seven tagSNPs in the *IRF1* gene. The genotype distributions of the seven studied tagSNPs were in Hardy-Weinberg equilibrium. Genotyping rate was larger than 96%, indicating reliability of genotyping. Logistic regression analysis revealed none of the studied tagSNPs of the *IRF1* gene was associated with TB risk (Table 3).

A strong linkage disequilibrium between SNP pairs was defined as r^2^>0.85. Figure 1 showed that, in the *IRF1* gene, rs2070724, rs2549007 and rs2706386 (r^2^>0.85), and rs2549007 and rs2706386 (r^2^ = 0.91) had a strong linkage disequilibrium.

### Relationship between tagSNPs of the *IRF8* Gene and TB

Table 4 showed the primary information for the fifteen tagSNPs in the *IRF8* gene. The genotype distributions of the fifteen studied tagSNPs were in Hardy-Weinberg equilibrium (all *P*>0.05) among the subjects except for rs424971 (*P*<0.01). The linkage disequilibrium between tagSNP pairs was shown in Figure 2. According to r^2^ value, the fourteen tagSNPs could be represented for the whole gene.

Logistic regression analysis revealed that three tagSNPs (rs925994, rs11117415 and rs10514611) in the *IRF8* gene were significantly associated with TB. The other tagSNPs were not significantly associated with TB (Table 5).

Akaike’s information criterion (AIC) is a measure of the goodness of fit of an estimated statistical model and it can judge a model by how close its fitted values tend to be to the true values in terms of a certain expected value. In the case of rs925994, the log-additive model was accepted as the best fit for these data because of the smaller AIC value. It had an association of 1.75 (1.35∼2.27) with TB, indicating that an 75% increase in risk associated with each additional copy of the A allele of rs925994 (*P = *0.002 with Bonferroni correction for 22 SNPs). When rs11117415 exhibited a co-dominant effect, patients with genotypes AG and GG had a 1.44-fold (OR = 1.44, 95% CI 1.09∼1.91) and 4.75-fold (OR = 4.75, 95% CI 2.16∼10.43) increase in risk compared to patients with genotype AA, respectively (*P = *0.002 with Bonferroni correction for 22 SNPs). When rs10514611 exhibited a recessive effect, patients with genotype TT had a 3.39-fold (OR = 3.39, 95% CI 1.60∼7.20) increase in risk compared to patients with genotype CC and TC (*P = *0.015 with Bonferroni correction for 22 SNPs).

### Functional Analysis of rs10514611 by Reporter Gene Assay

MicroRNAs are small noncoding transcripts of ∼22 nucleotides that inhibit the translation or promote the degradation of complementary mRNA, usually by binding to the 3′-UTR of the gene. Analysis using PolymiRTS (http://compbio.uthsc.edu/miRSNP) indicated that rs10514611 was located within a predicted binding site of miR-330 (Table S3). We therefore examined whether rs10514611 of the *IRF8* gene could be a target for microRNA.

To confirm the functional significance of rs10514611, we performed a reporter assay using reporter constructs containing the *IRF8* 3′-UTR. We inserted the *IRF8* 3′-UTR that included either the rs10514611 C allele or the rs10514611 T allele into the 3′-UTR region of the pMIR-REPORT vector.

When the reported vectors were transfected into the HeLa cell line, the construct that contained the non-risk allele (rs10514611 C) showed significantly higher luciferase activity than did the risk T allele (*P*<0.01) (Fig. 3). These results indicated that rs10514611 that was associated with relative risk to TB, in particular when occurring homozygously as TT genotype, might change the expression of IRF8 protein which played an important role in resistance to intracellular infection.

## Discussion

IRF1 and IRF8 play an important role in the pathogenesis of TB. However, the *IRF1* and *IRF8* gene polymorphisms have not been studied much in TB. In our present study, we conducted a case-control study consisting of 495 controls and 452 ethnically matched cases with TB in a Chinese population to investigate the relationship between them and TB susceptibility.

Most of the tagSNPs selection strategies are haplotype-based. Therefore, these tagSNPs are informative polymorphisms that best characterize the haplotype diversity of a given chromosomal region [24]. A series of studies show that it is possible to retain much of the information of haplotypes by retaining only a reduced subset of markers (tagSNPs) [25], [26]. Therefore, we used tagSNPs as marker to investigate the relationship between the polymorphisms of the *IRF* gene family and TB susceptibility.

Here, we studied seven tagSNPs of the *IRF1* gene and TB risk in a case-control study of a Chinese population by using the Environmental Genome Project database (http://egp.gs.washington.edu). Among the seven tagSNPs, rs2549007 is notable in that it is located in the 5′ upstream region containing the NF-κB binding site, and it has been shown to have higher transcriptional activity [20], [27]. The positional effect has been associated with susceptibility to atopy [20]. Lee *et al.* reported that rs2549007 and the haplotype formed by this SNP, and other adjacent SNPs, including rs2706384, rs2549009 and rs839, had a close relationship with Behcet’s disease [21]. Schedel *et al.* reported that rs2070721, rs41525648 and rs2070729 was associated with hereditary allergies [20]. Mangano *et al.* found that rs2070724 was related with plasmodium infections [22]. All the data inferred that the seven tagSNPs were related to some infectious disease. However, we have not found an association between the tagSNPs of the *IRF1* gene and TB in the present study. Vollstedt *et al.* (2009) also reported no association of the *IRF1* gene with TB in the two Southeast Asian populations (Indonesian and Vietnamese) as well [23], which was in accordance with our results. However, the relationship between the *IRF1* gene and TB should be performed in large number of cases and controls in different populations. Furthermore, rs2549007 (MAF: 0.34) and rs2549008 (MAF:0.11) in the promoter region of the *IRF1* gene existed among a Chinese population, which provided additional SNP information for *IRF1* gene.

IRF8 is a member of the interferon regulatory factors that has a pivotal role in mediating resistance to pathogenic infections and in promoting the differentiation of myeloid cells [16]. Loss-of-function mutation in the mouse *IRF-8* gene may impair the induction of type I IFN, which result in rapid dissemination of the infection of mycobacterium TB and rapid necrosis of infected tissues [16]. Furthermore, IRF8 modulates TLR signaling and may contribute to the crosstalk between IFN-γ and TLR signal pathways, thus acting as a link between innate and adaptive immune responses [28]. In the present study, we analyzed fifteen tagSNPs of the *IRF8* gene in Han Chinese population and we identified three of them (rs925994, rs11117415 and rs10514611) were associated with susceptibility to TB. Per allele OR was 1.75 (95% CI 1.35∼2.27, *P* = 0.002), 4.75 (95% CI 2.16∼10.43, *P* = 0.002), and 3.39 (95% CI 1.60∼7.20, *P* = 0.015) respectively. All these findings indicated that the *IRF8* might be involved in the pathogenesis of TB through genetic mechanisms.

Another new finding of this study was that rs10514611 was located in the binding region of microRNA miR-330, according to bioinformatics analyses. Transfection experiments showed that miR-330 could inhibit the expression of a reporter construct containing the risk allele (rs10514611 T). The base substitution of rs10514611 might affect the base-pairing between the miR-330 and its target site, and hence the expression of the risk allele was downregulated by miR-330. These results suggested a potential mechanism of down-regulated expression of the *IRF8* in the carriers of the risk allele. Indeed, the absence of IRF8 expression impairs killing of intraphagosomal *Mycobacterium bovis* in mice [29]–[31]. IRF8 expression also induces the expression of interleukin 12 [32]. Thus it can be seen that downregulation of IRF8 expression might be the reason for rs10514611 (T)’s association with susceptibility to TB. In addition, these investigations of miR-330 expression pattern might provide new insight into the role of microRNA in TB.

However, there were three limitations regarding the present study. One limitation was about the sample size. Increasing the sample size may prove helpful and/or necessary in order to increase power to detect underlying susceptibility genes or loci. Another limitation could be due to correction of multiple genetic model and the method of multiple testing. The Bonferroni correction for mutliple SNPs was overly conservative, it might be give rise to a false-negative rate in association studies. The third limation was sample heterogeneity because about 39.4% of the cases were clinically diagnosed without microbiologic confirmation. Therefore, Association of the *IRF8* gene with TB should be tested for reproducibility to validate the role of the *IRF8* in TB occurrence.

In conclusion, the present study suggested that genetic variants in the *IRF8* gene was associated with risk of TB. The tagSNPs rs925994, rs11117415, and rs10514611 in the *IRF8* gene had a strong association with TB risk and the *IRF8* might emerge as new and attractive molecular target in TB.

## Supporting Information

Table S1 **Primer sequences used for amplification of the** ***IRF1*** **and** ***IRF8*** **genes.**
(DOC)

Table S2 **Oligonucleotide extension primer sequences used for the** ***IRF1*** **and** ***IRF8*** **gene.**
(DOC)

Table S3 **SNPs in miRNA target sites.**
(DOC)
