# Supplementary material for: Tagging Single Nucleotide Polymorphisms in the IRF1 and IRF8 Genes and Tuberculosis Susceptibility
Source: PLoS One. 2012 Aug 6;7(8):e42104. doi: 10.1371/journal.pone.0042104 (PMC3412841; doi:10.1371/journal.pone.0042104)
Supplement: Table S1 — Primer sequences used for amplification of the IRF1 and IRF8 genes. (DOC) [file pone.0042104.s001.doc]

**Table S1 Primer sequences used for amplification of the *IRF1* and *IRF8*** genes

| Gene | SNP number | Primer sequence | Fragment |
| --- | --- | --- | --- |
| *IRF1* | rs2070721 | Sense: 5’-CCCAGTGCTCCCGGCTTT-3’ | 89 |
|  |  | Antisense: 5’-AGTAAGCCAGCCCTTGCC-3’ |  |
|  | rs2070724 | Sense: 5’-ATGTCTTGCAAACTAAGAAAGCA-3’ | 95 |
|  |  | Antisense: 5’-TCAGGGAGAGTGCTGCTG-3’ |  |
| *IRF8* | rs10514611 | Sense: 5’-TTAACTATCATTTCCAAAGACTTGTC-3’ | 101 |
|  |  | Antisense: 5’-AACCTTGTTTTCACAAGTTGATTT-3’ |  |
|  | rs6638 | Sense: 5’-TTGTCCCTTTTGTCCAACA-3’ | 90 |
|  |  | Antisense: 5’-AGATTCAAGCCTATTGTTATCTTATGA-3’ |  |
|  | rs305080 | Sense: 5’-AATGGGATCGGGATGTCC-3’ | 112 |
|  |  | Antisense: 5’-AACTAGGAGGTAACCACAGTCAGA-3’ |  |
|  | rs4843860 | Sense: 5’-ACAAGGCAGCCTCGAACC-3’ | 151 |
|  |  | Antisense: 5’-AATCTAAGGTAGGAATGCAGATAACA-3’ |  |
|  | rs16939967 | Sense: 5’-ATGCAGAGGCACAGTTTGG-3’ | 111 |
|  |  | Antisense: 5’-AGAGGTACCAAAGCTCACAGC-3’ |  |
|  | rs11117415 | Sense: 5’-CGTGAATGGTGGGTTGTC-3’ | 94 |
|  |  | Antisense: 5’-ATTCCCTGCTGACACTGCT-3’ |  |
|  | rs1044873 | Sense: 5’-TTCATTTCTGTCATGAAATCATTC-3’ | 145 |
|  |  | Antisense: 5’-ATTGGTGCTGACTCAGGCT-3’ |  |
|  | rs2292980 | Sense: 5’-AAATTCTGTGCCTTTCCCAA-3’ | 90 |
|  |  | Antisense: 5’-CTGAAGCCATAATCTACTCTGTAAATG-3’ |  |
|  | rs424971 | Sense: 5’- TCAGAGCTTGTCTGCGTGT -3’ | 100 |
|  |  | Antisense: 5’- AATAAGTTTCYTGGCCCTTTC-3’ |  |
|  | rs925994 | Sense: 5’- TTACATTCTGTTATCTTCATTCTCTGA -3’ | 155 |
|  |  | Antisense: 5’- TATTACAAGGTGGACATTTGCA -3’ |  |
