# Supplementary material for: Tagging Single Nucleotide Polymorphisms in the IRF1 and IRF8 Genes and Tuberculosis Susceptibility
Source: PLoS One. 2012 Aug 6;7(8):e42104. doi: 10.1371/journal.pone.0042104 (PMC3412841; doi:10.1371/journal.pone.0042104)
Supplement: Table S2 — Oligonucleotide extension primer sequences used for the IRF1 and IRF8 gene. (DOC) [file pone.0042104.s002.doc]

**Table S2 Oligonucleotide extension primer sequences used for the *IRF1* and *IRF8* gene**

| Gene | SNP number | Primer sequence (5’→3’) | Sequence of extension product (5’→3’) |
| --- | --- | --- | --- |
| *IRF1* | rs2057656 | Sense: 5’- aaggccaaggactgagagtgttatga -3’  Antisense: 5’-tagtcagaggccacagggcttg -3’ | 5’-TTTTTTGGCCCCTCCACACATCC-3’ |
|  | rs2706381 | Sense: 5’-TCCTCAGGCATCCTCAGGCATA-3’  Antisense: 5’- ccaggaCAGACCGAGctgaaag-3’ | 5’-TTTTTTTTTTTTTTTTTTTTTTTGAAAGGACCTCTCTGATAGTGGC-3’ |
|  | rs2706386 | Sense: 5’- CGGGTAGGGGAGGGCTTG-3’  Antisense:5’-GCGTTTGCAAGAAACCGAAACC-3’ | 5’-CCCCATCGCCGGGTTCCG-3’ |
|  | rs2549007 | Sense: 5’-TATCCCACTGGCAagcttgagg-3’  Antisense: 5’- tgtcctctcACTCCGCCTTGTC -3’ | 5’-TTTTTTTTTTTTTTTTTTTTTTTTTTTTTTTTTTTTTTTTTTTGTGCCCGGGCGATCCCCTC-3’ |
|  | rs2549008 | Sense: 5’- TATCCCACTGGCAagcttgagg -3’  Antisense: 5’- tgtcctctcACTCCGCCTTGTC -3’ | 5’-TTTTTTTTTTTTTTTTCTGCGTTCGGGAGATATACC-3’ |
| *IRF8* | rs12929551 | Sense: 5’-TAGGATGGCGTCTCCcttctcc -3’  Antisense: 5’- GCTTAtgcccctgagctcacac -3’ | 5’-TTTTTTTTTTTTACACTATCTCGGGCTGTGCT -3’ |
|  | rs8064189 | Sense: 5’- tccttttgttatgaatgtaggcagca -3’  Antisense: 5’- TGGGGAAATCCATGTACTGCTtg -3’ | 5’-TTTTTTTTTTTTTTTTTTTTTTTTTTTTCAGTGAAGAAGTACAGAGAACACTGT -3’ |
|  | rs9926411 | Sense: 5’- tccttttgttatgaatgtaggcagca -3’  Antisense: 5’- TGGGGAAATCCATGTACTGCTtg -3’ | 5’-TTTTTTTTTTTTTTTTTTTTTCTTATGACAATACTCCGTCTCA -3’ |
|  | rs12924316 | Sense: 5’-acGGCACGGACGCAGTATTCTA -3’  Antisense: 5’- CAACCACAGTGAACAGCTCCAGTT -3 | 5’-TTTTTTTTTTTTTTTTTTTTAGCCCAAGACTGGAAGGAGA -3’ |
|  | rs182511 | Sense: 5’-CCCTTGGTCCTAACTCCAGAATAGC -3’  Antisense: 5’- TCCCAGAGGTGAAACCTGGACA -3’ | 5’-TTTTTTTTTTTTTTTTTTTTTTTTTTTTTTTTTTTTTGGACTCTGATGACCCACAAGG -3’ |
