# Supplementary material for: Tagging Single Nucleotide Polymorphisms in the IRF1 and IRF8 Genes and Tuberculosis Susceptibility
Source: PLoS One. 2012 Aug 6;7(8):e42104. doi: 10.1371/journal.pone.0042104 (PMC3412841; doi:10.1371/journal.pone.0042104)
Supplement: Table S3 — SNPs in miRNA target sites. (DOC) [file pone.0042104.s003.doc]

**Table S3** SNPs in miRNA target sites

| SNP ID | Ancestral Allele | Allele | miR ID | miRSite |
| --- | --- | --- | --- | --- |
| rs10514611 | C | C |  |  |
|  |  | T | Hsa-miR-330-3p | cTGCTT**T**GAtaaa |
